# PCR confirmation of infection with Cyclospora cayetanensis.

**DOI:** 10.3201/eid0204.960415

**Published:** 1996

**Authors:** N J Pieniazek, S B Slemenda, A J da Silva, E M Alfano, M J Arrowood


persons and the public health relevance of this emerging pathogen as a potential cause of diarrheal outbreaks (3,4) make prompt disclosure of the epidemiologic features and behavior of the parasite necessary. As we propose the possible participation of poultry in the epidemiologic cycle of the coccidia, we invite other *Cyclospora* working groups worldwide to confirm the so far putative reservoir described in this communication and to further study other possible hosts or reservoirs.

**H. Leslie García-López, Luís E. Rodríguez-Tovar, and Carlos E. Medina-De la Garza***
Facultad de Medicina y Hospital Universitario "Dr. J.E. González," Universidad Autónoma de Nuevo León, Monterrey, Mexico

### References

1. Ashford RW. Ocurrence of an undescribed coccidian in man in Papua New Guinea. Ann Trop Med Parasitol 1979;73:497-500.
2. Ortega YR, Gilman RH, Sterling CR. A new coccidian parasite (Apicomplexa: Eimeriidae) from humans. J Parasitol 1994;80:625-9.
3. Soave R, Johnson WD. *Cyclospora:* conquest of an emerging pathogen (commentary). Lancet 1995;345:667-8.
4. Huang P, Weber JT, Sosin DM, Griffin PM, Long EG, Murphy JJ, et al. The first reported outbreak of diarrheal illness associated with *Cyclospora* in the United States. Ann Intern Med 1995;123:409-14.
5. Rabold JG, Hoge CW, Shlim DR, Kefford C, Rajah R, Echeverría P. Cyclospora outbreak associated with chlorinated drinking water. Lancet 1994;344:1360.
6. Zerpa R, Uchima N, Huicho L. *Cyclospora cayetanensis* associated with watery diarrhoea in Peruvian patients. J Trop Med Hyg 1995;98:325-9.
7. Relman DA, Schmidt TM, Gajadhar A, Sogin M, Cross J, Yoder K, et al. Molecular phylogenetic analysis of *Cyclospora*, the human intestinal pathogen, suggests that is closely related to *Eimeria* species. J Infect Dis 1996;173:440-5.
8. Chiodini PL. A "new" parasite: human infection with *Cyclospora cayetanensis*. Trans Roy Soc Trop Med Hyg 1994;88:369-71.
9. Connor BA, Shlim DR. Foodborne transmission of cyclospora . Lancet 1995;346:1634.

### PCR Confirmation of Infection with *Cyclospora cayetanensis*

**To the Editor:** *Cyclospora cayetanensis*, formerly known as cyanobacterium-like body, is a variably acid-fast microorganism. Recently, it was classified as a coccidian parasite (1) closely related to the genus *Eimeria* (2). Humans infected with *C. cayetanensis* typically have diarrheal illness with a variable number of stools per day and sometimes have nausea and vomiting (3,4). *Cyclospora* infection has been reported in many parts of the world as clustered or sporadic cases (1,3-5).

Variable success in diagnosing infection with this parasite underscores the need for using (as quality control) molecular methods, which do not rely on the level of expertise of laboratory personnel in microscopy. The key features for diagnosis by light microscopy are size (8µm to 10µm in diameter), internal features of stained and unstained oocysts, and autofluorescence of oocysts (1,6). The definitive diagnosis is understood as visualization of characteristic sporulated oocysts, which contain two sporocysts. However, sporulation typically requires incubating oocysts for up to 2 weeks, and this approach cannot be applied to Formalin or polyvinylalcohol-preserved stool smears.

Sporadic and clustered cases of *Cyclospora* infections were reported in the United States and Canada during May and June 1996 (5,7). From these outbreaks, more than 900 cases were diagnosed by examining stool specimens under light microscopy (Barbara Herwaldt, pers. comm.). Epidemiologic studies indicated risk for *Cyclospora* infection from consuming raspberries imported from Guatemala (7). Forty-two stool specimens supplied in 2.5% potassium dichromate from patients with intestinal symptoms were forwarded to the Centers for Disease Control and Prevention to be evaluated by microscopy and by polymerase chain reaction (PCR) amplification. In addition, one well-characterized positive stool specimen from Nepal was provided by John Cross, Armed Forces Research Institute of Medical Sciences,

Bangkok, Thailand, to use as the positive control.

Using techniques we developed for diagnosis of other protozoan parasites in stools, we extracted DNA from all stools. The techniques we used employ glass-bead disruption of oocysts in a buffer containing Laureth-12, purification with the RapidPrep Micro Genomic DNA Isolation Kit for Cells and Tissue (Pharmacia Biotech Inc., Piscataway, N.J.), followed by a final purification step employing the QIAquick PCR purification kit protocol (Qiagen, Inc., Chatsworth, Calif.) (8). The glass-bead disruption of oocysts was far more effective than sonication (2) or freeze-thawing techniques (9). We performed nested PCR in all stool specimens by using Relman et al. (2) primers CYCF1E and CYCR2B for the first step of nested amplification and primers CYCF3E and CYCR4B for the second (nested) step of the PCR. These are the only primers described for amplification of *Cyclospora* DNA. We found optimal conditions for the first step PCR to be denaturation at 94°C for 30 s, annealing at 55°C for 30 s, and extension at 72°C for 90 s, 45 cycles. The same conditions were used for the second step of the nested PCR, but the annealing temperature was 60°C.

By using this approach, we amplified the *Cyclospora*-specific DNA fragment in 16 (38%) of the 26 (62%) specimens reconfirmed as positive by light microscopy. The 10 specimens negative by PCR but positive by microscopy showed either few or moderate numbers of *Cyclospora* oocysts. None of the 16 (38%) specimens negative by microscopy generated positive results in the PCR *Cyclospora* test. Upon further examination by the PCR technique we developed (9), three of these samples were positive for another enteric coccidian, *Cryptosporidium parvum*.

Preliminary evaluation indicates that the sensitivity of PCR is 62%, and the specificity is 100%. Although the sensitivity of the technique should be evaluated further, these results indicate that PCR can be used to detect *Cyclospora*. We assessed the sensitivity of this PCR again by using the Nepalese specimen described above. This specimen, which was used as positive control in all reactions, was amplified even when the extracted DNA was diluted at $10^{-5}$.

Lastly, a note of caution. As noted by Relman et al. (2) and confirmed by us through GenBank searches, the nested PCR *Cyclospora* primers cross-amplify other coccidians, especially those belonging to the genus *Eimeria* (because no molecular data exist for another human coccidian enteric parasite, *Isospora belli*, potential cross-amplification remains to be determined). This cross-amplification with *Eimeria* should not present a problem in diagnosing *Cyclospora* in human stool, as no human infections by *Eimeria* are known. However, when analyzing food or environmental specimens, this cross-amplification may complicate precise detection of *Cyclospora*.

**Norman J. Pieniazek, Susan B. Slemenda, Alexandre J. da Silva, Edith M. Alfano, and Michael J. Arrowood**
Centers for Disease Control and Prevention, Atlanta, Georgia, USA

### References

1. Ortega YR, Sterling CR, Gilman RH, Cama VA, Diaz F. *Cyclospora cayetanensis*: a new protozoan pathogen of humans. N Engl J Med 1993;328:1308-12.
2. Relman DA, Schmidt TM, Gajadhar A, Sogin M, Cross J, Yoder K, et al. Molecular phylogenetic analysis of *Cyclospora*, the human intestinal pathogen, suggests that it is closely related to *Eimeria* species. J Infect Dis 1996;173:440-5
3. Soave R, Dubey JP, Ramos LJ, Tummings M. A new intestinal pathogen? Clin Res 1986;34:533A.
4. Long EG, Ebrahimzadeh A, White EH, Swisher B, Callaway CS. Alga associated with diarrhea in patients with aquired immunodeficiency syndrome and in travelers. J Clin Microbiol 1990;28:1101-4.
5. Centers for Disease Control and Prevention (CDC). Outbreaks of *Cyclospora cayetanensis* infection - United States, 1996. MMWR Morb Mortal Wkly Rep 1996;45:549-51.
6. Garcia LS, Bruckner DA. Intestinal protozoa: Coccidia and microsporidia. In: Garcia LS, Bruckner DA, editors. Diagnostic medical parasitology, 2nd ed. Washington: American Society for Microbiology, 1993;49-74.
7. Centers for Disease Control and Prevention (CDC). Update: Outbreaks of *Cyclospora cayetanensis* infection - United States and Canada, 1996. MMWR Morb Mortal Wkly Rep 1996;45:611-2.
8. da Silva AJ, Schwartz DA, Visvesvara GS, de Moura H, Slemenda SB, Pieniazek NJ. Sensitive PCR diagnosis of infections by *Enterocytozoon bieneusi* (microsporidia) using primers based on the region coding for small-subunit rRNA. J Clin Microbiol 1996;34:986-7.

9. Johnson DW, Pieniazek NJ, Griffin DW, Misener L, Rose JB. Development of a PCR protocol for sensitive detection of *Cryptosporidium* oocysts in water specimens. Appl Env Microbiol 1995;61:3849-55.

### Emerging Infectious Diseases and the Depopulation of French Polynesia in the 19th Century

**To the Editor:** The same dynamics now considered factors in the emergence of infectious diseases may have been involved in the dramatic depopulation of French Polynesia in the 19th century. Temporal and geographic variation in the frequency and severity of infectious diseases are the result of the encounter and interaction of a population of parasites and a population of hosts. J. Musser reviewed the “bacterial side of the equation” (1). On the host side, there are two historical models that describe the influence of parasitism on human populations (2-4): 1) the South American model, in which new pathogens were introduced into native populations by the European conquistadores, causing the death of 50 million people; and 2) the African model, in which infectious diseases present in native populations protected them from the effects of colonization until modern times when the discovery of quinine and other efficient antipathogenic drugs provided added protection. The second model is well illustrated by the attempted colonization of Madagascar, where the French lost five men to war and 5,000 to malaria (2). This letter intends to illustrate the first model. We suggest that during their first contacts with European navigators in the very late 18th century and the 19th century, Polynesian islanders, much like populations in the South American model, were decimated by newly introduced infectious diseases.

It is difficult to know precisely which infectious diseases were present in Tahiti and the other French Polynesian islands before the arrival of the first Europeans. However, a study of Polynesian languages indicates that Bancroftian filariasis and leprosy were already present, while syphilis and other venereal diseases, influenza, and tuberculosis (TB) were probably unknown. Epidemic diarrhea and dysenteriae could have existed, although first reports mentioned that the oldest Polynesians “never heard of dysenteriae before” (5). In the Marquesian language, names exist for leprosy, bronchitis, abscesses, and impetigo.

The number of inhabitants in Tahiti, as well as in the Marquesas and the Austral Archipelago, was at first only estimated by European explorers. However, a precise census was performed as soon as missionaries and French authorities noted the high death rates in most of the islands (5,7,15,16). Tahiti was annexed by France in 1843; the first census was performed in 1848, and the population size was assessed approximately every 5 years until 1911.

Four major epidemic diseases (TB, typhoid, influenza, and smallpox) devastated the Marquesas from 1791 to 1863/64; approximately 80% of the population died. During that period, exchange of populations between the Marquesas Islands also increased, as a consequence of colonization. Thus, leprosy increased dramatically during the second half of the 19th century, to a prevalence of 4.11% in 1884 (6).

In Rapa, the remote, southern island of the Austral Archipelago, at least three epidemics were reported, resulting in the loss of more than 90% of the population. Although the cause of the first epidemic remained unknown, dysenteriae and smallpox were identified as causes of the second and third epidemics, respectively.

From Rapa, a missionary went to Mangareva in 1831 or 1832, and his visit there was followed by an epidemic that the natives attributed “to his god.” He had to flee back to Rapa. The second recorded epidemic disease was “Chinese scabies” in 1865, which decimated the child population. Then, the warship “La Zélée” brought an epidemic of influenza in 1908. In 1910, TB and leprosy were reported “to spread rapidly” (7), and in 1911, the ship “La Gauloise” brought whooping cough to Mangareva.

In Tahiti and the Society Islands, the number and diversity of international and interisland exchanges, involving numerous commercial ships and whalers, make the origin of epidemics more difficult to trace.

